# Spatiotemporal receptive field properties of epiretinally recorded spikes and local electroretinograms in cats

**DOI:** 10.1186/1471-2202-6-50

**Published:** 2005-08-15

**Authors:** Marcus Wilms, Reinhard Eckhorn

**Affiliations:** 1 Institute of Neurophysics, Philipps-University Marburg, Renthof 7, 35032 Marburg, Germany; 2 Institute of Medicine, Research Centre Jülich, 52425 Jülich, Germany

## Abstract

**Background:**

Receptive fields of retinal neural signals of different origin can be determined from extracellular microelectrode recordings at the inner retinal surface. However, locations and types of neural processes generating the different signal components are difficult to separate and identify. We here report epiretinal receptive fields (RFs) from simultaneously recorded spikes and local electroretinograms (LERGs) using a semi-chronic multi-electrode *in vivo *recording technique in cats. Broadband recordings were filtered to yield LERG and multi unit as well as single unit spike signals. RFs were calculated from responses to multifocal pseudo-random spatiotemporal visual stimuli registered at the retinal surface by a 7-electrode array.

**Results:**

LERGs exhibit spatially unimodal RFs always centered at the location of the electrode tip. Spike-RFs are either congruent with LERG-RFs (N = 26/61) or shifted distally (N = 35/61) but never proximally with respect to the optic disk. LERG-RFs appear at shorter latencies (11.9 ms ± 0.5 ms, N = 18) than those of spikes (18.6 ms ± 0.4 ms, N = 53). Furthermore, OFF-center spike-RFs precede and have shorter response rise times than ON-center spike-RFs. Our results indicate that displaced spike-RFs result from action potentials of ganglion cell axons passing the recording electrode *en route *to the optic disk while LERG-RFs are related to superimposed postsynaptic potentials of cells near the electrode tip.

**Conclusion:**

Besides contributing to the understanding of retinal function we demonstrate the caveats that come with recordings from the retinal surface, i.e., the likelihood of recordings from mixed sets of retinal neurons. Implications for the design of an epiretinal visual implant are discussed.

## Background

The intact retina of animals and humans provides several sources of electrical responses to visual stimulation. Among them, the receptors, horizontal, bipolar, and to some extent the amacrine cells generate graded local potentials [1]. Their different superimposed neuronal contributions have extensively been studied in the electroretinogram (ERG) [2-6]. The retinal output comprises ganglion cells that possess functional specificity in that they preferentially respond to visual stimuli with contrasting center and surround structure flashed onto a restricted area of the retinal surface called the receptive field (RF) [7]. Ganglion cell spikes travel along axons beneath the inner retinal surface to the cortical centers for further visual processing. Interestingly, the axonal trajectory [8] and layering in the retina is highly organized. Radius & Anderson [9] reported that in primates axons from more central ganglion cells lie more superficially than axons arising from more peripheral retinal sites. Minckler [10] added to this that axonal bundles are consistently organized in a way that axons of peripapillary origin lie most superficially. It is because of this superficial arrangement of axons that the interpretation of epiretinal electrical recordings should meet necessary caveats. Thus, when Kuffler [7] performed his pioneering epiretinal electrical recordings in the cat retina, he distinguished axon from ganglion cell potentials by the distinct polyphasic waveforms and shifted RFs of axon potentials. He did not, however, further investigate the spatial relationships between the RFs of different spike sources and he did not record the local ERG response. Other studies used intracellular recordings from optic tract (e.g., [11]) or the cornea (e.g., [12]) lack the spatial resolution needed for revealing contributions of distinct retinal signal sources. Chichilnisky & Kalmar [13] registered spikes *in vitro *in macaque monkey retina but constrained the angular extent of the visual stimulus for the sake of spatial resolution. This may have obscured the contributions of shifted axonal spike-RFs. Recently, Segev and coworkers [14] used multi-electrode array and spike-sorting techniques to record retinal signals from a large fraction of ganglion cells in a patch of *in vitro *salamander retina. They mostly recorded somatic spikes and reported only rare observations of axonal spikes presumably due to the arrangement of axons in tight bundles. The few axonal RFs they observed were displaced with respect to the somatic RFs. We here present data of broadband epiretinal recordings in *in vivo *cat retina that allowed us to compare the spatio-temporal RFs of distinct spike sources and LERG signals measured simultaneously with the same microelectrode.

The superficial arrangement of ganglion cell axons is also expected to play a role in the design of an epiretinal visual implant [15,16]. Epiretinally applied electrical stimuli can activate axons as well as somata which should potentially lead to non-retinotopic cortical activations. This poses the question whether a useful epiretinal implant is feasible. One way to test this is to intracortically record evoked potentials in response to epiretinal electrical stimuli [17]. However, this approach is not optimal as it is not known in advance where exactly the cortical activation following an epiretinal axon stimulation is to be expected, making it difficult to access the right cortical site with a microelectrode. Another way of approaching the issue of retinotopic mapping is from the perspective of the receptive field concept. The latter is fundamental for the understanding of how the outside visual world retinotopically maps to neurons of the visual pathway. The analysis of retinal rather than cortical receptive fields allows one to specifically ask which retinal neurons might be activated by epiretinal stimuli. This makes sense assuming that a similar group of retinal neurons can be stimulated as well as recorded from by the same epiretinal microelectrode. We thus can avoid electrical stimulation tests in human patients or animals by detecting the neuronal structures a recording electrode is sensitive to, rather than the neuronal structures the same electrode excites when being switched to stimulation mode. We argue why this indirect approach is reasonable and discuss possible implications for the design and resolution of a visual prosthesis that is based on epiretinal electrical stimulation [17].

## Results

We present data from experiments in four adult cats (experiments *140*, *302*, *and 033*: right eye; *142*: left eye). Each set of data was recorded using seven epiretinal microelectrodes simultaneously in one or more electrode array positions per experiment. RF characteristics for each electrode position were typically confirmed more than once but they only once contribute to the present study resulting in a total of 44 different retinal recording positions.

### Spatial RF aspects

**Figure 1:**
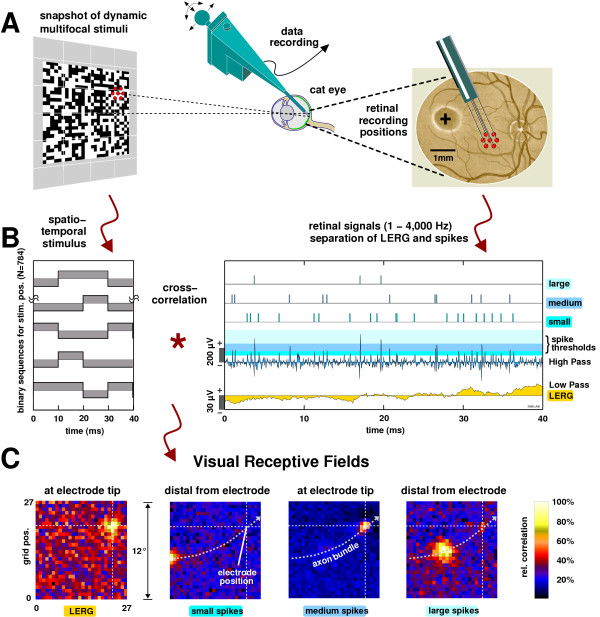
**Setup for the analysis of retinal RFs. A: **The dynamic multifocal visual stimulus was presented on a computer monitor 1.3 m in front of the cat. Retinal signals were recorded by epiretinally positioned electrodes. **B: **Spikes were extracted from high-pass filtered (0.5–1 kHz) broadband data using an amplitude thresholding approach. LERG and spike signals were cross-correlated with the binary stimulus sequences at all pixel positions (N = 784). **C: **Example RF maps for the four signals separated from one electrode recording shown in B. White crosshairs indicate the back-projected epiretinal electrode positions that served as reference points for the RF centers. Crosscorrelation values for a 25 ms time delay between stimulus and response are color-coded for each signal separately (dark blue: weak or negative correlation, light yellow: strong positive correlation). The polarity of the LERG-RF is reversed to ease its comparison to the spike-RFs. Note that in this example the neurons responsible for LERG and the *medium sized *spikes both have RF centers that are congruent with the position of the electrode tip. The neurons responsible for *small *and *large sized *spikes, however, have RF centers that are clearly shifted by several degrees of visual angle. All RFs are of ON-type in this example. (Data from experiment *302*, right eye)

**Figure 2:**
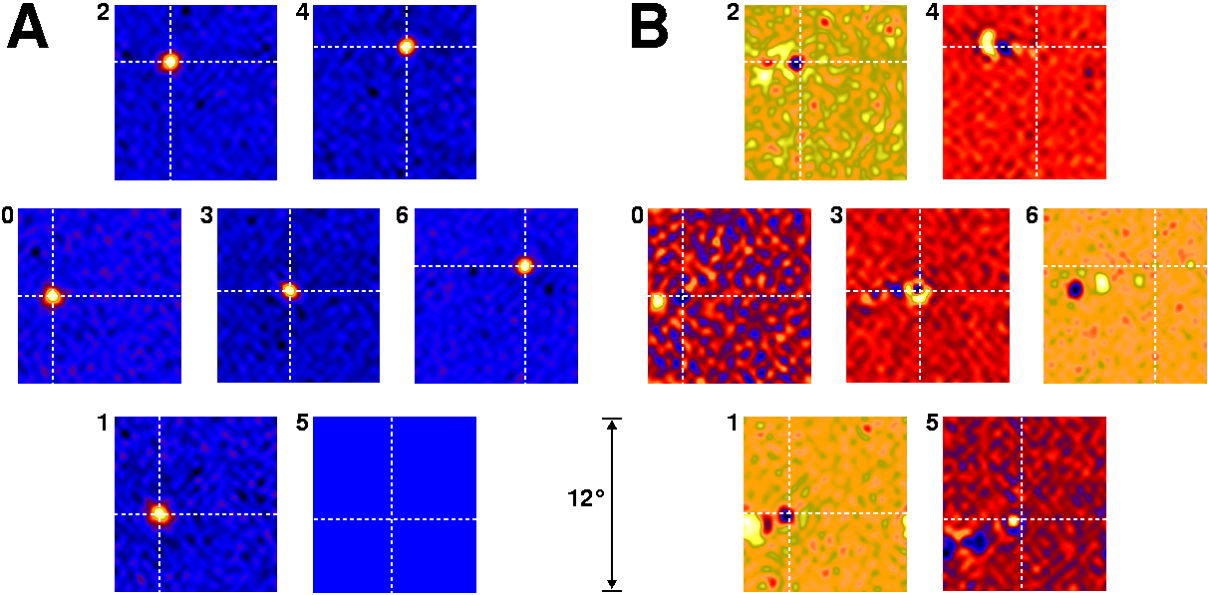
**Retinal RFs of simultaneously recorded LERG and MUA. **Simultaneously recorded retinal LERG- (**A**) and MUA-RFs (**B**). Individual RF maps are interpolated and arranged according to the hexagonal electrode array configuration. **A**: All LERG-RFs are congruent with the projected electrode tip positions (white crosshairs). The LERG from electrode *5 *was not available in this recording due to an amplifier failure. **B**: The MUA-RF maps exhibit multiple spatially segregated peaks of positive or negative polarity that are shifted to the left from the projected electrode position. The optical disk is located in the upper right. Color coding as explained in Fig. 1. (Data from experiment *140*, right eye)

The receptive fields estimated for epiretinally recorded signals often exhibited multiple, spatially segregated peaks (e.g., Fig. 1C, Fig. 2). We found LERG-RFs at 34/44 (77 %) of the recording positions. In the remaining 10/44 (23 %) we could not evaluate LERG-RFs either due to low signal-to-noise ratio or due to electrode dysfunction. LERG-RFs were always spatially unimodal and located at the actual position of the electrode as verified by back-projecting the electrode tip to visual space. Figure 2A exemplifies this finding: Each RF map corresponds to one retinal electrode and is arranged according to the hexagonal electrode array configuration. For example, electrode *2 *was located at a retinal position corresponding to the upper left in visual space. Accordingly, the back-projection of the electrode tip (white crosshair) as well as the automated RF mapping (colored blob) points at an upper left position within the RF map. We were able to estimate RFs for six of the seven retinal LERG signals in this example, all being congruent with the projected electrode positions. The location of electrode *5 *was only mapped using our back-projection protocol together with manual RF mapping based on spike activity (cf. *Methods*).

Multi unit activity (MUA, cf. *Methods*) comprises spikes from several neurons and exhibits multiple RFs that were found to be aligned along the estimated course of fiber bundles at the respective retinal eccentricity. This can be seen in Fig. 2B where we show RFs of MUA recordings that were simultaneously recorded with the LERGs in Fig. 2A. Note the spatially segregated peaks of positive or negative polarity that are all shifted to the left from the projected electrode position. To test whether such peaks correspond to ON- or OFF-RFs of single units we separated the retinal spikes from the raw broadband data (cf. *Methods*). We found that spikes separated by an amplitude threshold criterion belong to functionally distinct ganglion cells that differ in RF center locations, polarities, and/or time courses. We detected 81 spike- (or MUA-) RFs at 34/44 (77 %) of the recording positions. Thus, on average, we found about 1.8 spike-RFs per retinal recording position.

**Figure 3:**
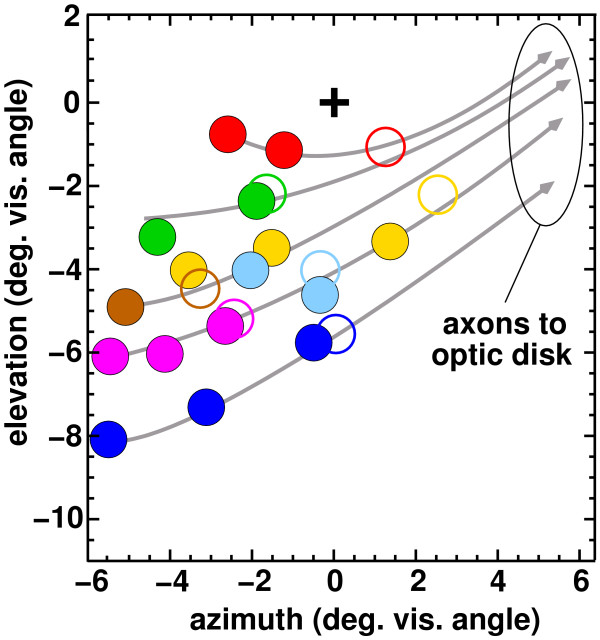
**Superimposed RF-centers for simultaneously recorded LERG and MUA. **Superimposed RF centers of the simultaneously recorded retinal LERG- (open circles) and MUA-RFs (filled circles) in Fig. 2. The black cross denotes the area centralis position. Gray arrows approximate the trajectories of ganglion cell axon bundles (reconstructed from [8]). Each color codes one retinal electrode. Note that MUA-RFs are shifted distally from the corresponding LERG-RFs with respect to the optic disk.

When LERG- and spike-RFs were detected simultaneously (in 28/44 (64 %) recording positions, 61 spike-RFs) we compared the relative position of RFs: Spike-RFs were either congruent with LERG-RFs (*local *RFs, i.e., located at the projected recording position; N = 26/61 (43 %)) or shifted distally (N = 35/61 (57 %)) but never proximally with respect to the optic disk. This strongly indicates that displaced spike-RFs result from the recording of spikes from passing axons *en route *to the optic disk. This is illustrated in Fig. 3 where we superimposed the LERG- and MUA-RFs from Figure 2 in addition to the estimated trajectories of ganglion cell axon bundles (reconstructed from [8]). MUA-RFs are either congruent with, or shifted distally from, the LERG-RFs with respect to the optic disk. Note that the positions of the LERG-RFs roughly resemble the hexagonal geometry of the electrode array. The geometry appears slightly distorted due to the angle at which the electrodes approached the retina and minor imperfections in the electrode array geometry.

Spike-RFs were found at all distal retinal separations from the retinal electrode tip that were mapped by the visual stimulus. They were either of ON-type (53/81; 65 %) or OFF-type (28/81; 35 %), whereas LERG-RFs were always of OFF-type (N = 34).

### Temporal RF aspects

Apart from the spatial aspects of retinal RFs we also analyzed their temporal characteristics to further elucidate the origin of LERG- and shifted spike-RFs. For this we plotted the averaged time course of the retinal response to stimulation within the respective RF center (this is the stimulus-response cross-correlation) and calculated the response latency (cf. *Methods*). Only the broadband recordings were appropriate (N = 3 experiments) as they provided sufficient temporal resolution for the detection of small latency differences. In one experiment we lost the exact temporal information about LERG due to wrong filter settings reducing the number of experiments with broadband recorded LERG to two (N = 18 LERG-RFs). Broadband recorded spikes were available in three experiments (N = 53 spike-RFs).

**Figure 4:**
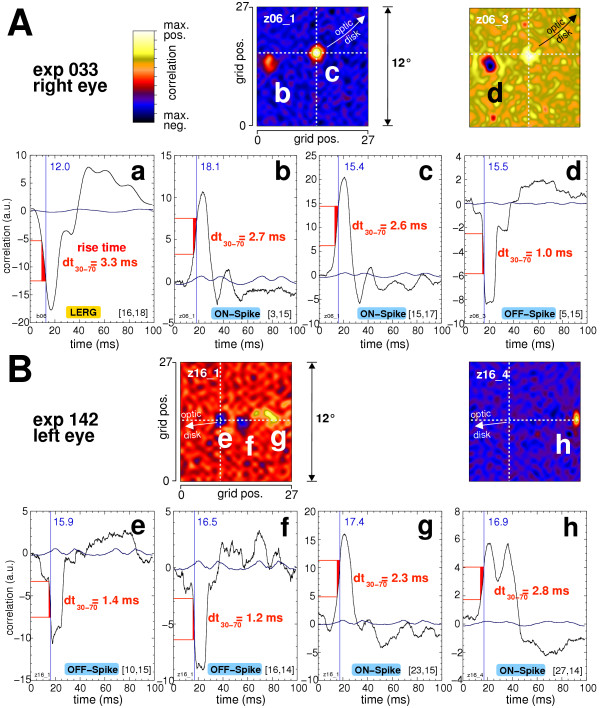
**Example RF time courses. **Examples for the different kinds of retinal RF time courses. **A, upper row: **Example spike-RFs for one retinal recording position in the right eye in experiment *033*. The two RF maps were calculated from spike trains derived from the same broadband recording. Note the ON- and OFF-center RFs that are slightly shifted with respect to each other and shifted distally from the projected electrode position (crosshair). **a-d: **Time courses of the retinal responses to stimulation with the optimal stimulus (i.e., in the center of the particular RF). From left to right: LERG (**a**), left RF of first spike train (**b**), right RF of first spike train (**c**), left RF of second spike train (**d**). Response rise times from 30%–70% of the peak amplitude (red) and latencies (blue) are indicated. The baseline (dark blue) is calculated from the average correlation strength between the retinal signal and all pixel luminance time courses. **B: **Similar results for recordings from the left eye in another experiment (*142*). Four spike-RFs and corresponding time courses are shown (**e-h**).

Typical data from two experiments are presented in Fig. 4, where LERG, spike-ON, and spike-OFF RFs are plotted along with the corresponding averaged time courses of the retinal responses to stimulation in the RF center. Figure 4A (upper row) shows RF maps for two retinal spike trains that were separated from the same and simultaneous electrode recording (experiment *033*, right eye). Both spike signals comprise two components as is evident from the two RFs in each map. The spatial shift of the spike-RFs with respect to the projected electrode tip position (crosshair) is to the left as the recording was made paracentrally in the right eye. The averaged time courses of the retinal signals in response to stimulation in their respective RF center (stimulus grid coordinates given in the lower right corners) are shown in Figure 4A (a-d). The average LERG response (leftmost graph) exhibits a smooth negative deflection with a short latency of 12.0 ms. For spike recordings ON-responses can be recognized by a peak of positive, OFF-responses by a peak of negative correlation with stimulus onsets. Interestingly, the spike ON-responses in the left and middle graphs evolve smoothly whereas the OFF-response depicted in the rightmost graph evolves in a stepwise fashion. In quantitative terms the ON-response latency and rise time is 18.1 and 2.7 ms in the left graph, and 15.4 and 2.6 ms in the middle graph. The longer latency in the left graph can partly be attributed to the longer conduction time for spikes that need to travel from their site of initiation (center of RF) to the recording position. The latency and rise time for the OFF-response is 15.5 and 1.0 ms.

Similar results can be derived from Figure 4B. Again RF maps are plotted for two separated spike populations of the same electrode recording (experiment *142*, left eye). Here the left spike-RF map comprises three components (two OFF and one ON) and the right map only one (ON) as is evident from the peaks in the correlation maps. The spatial shift of spike-RFs with respect to the projected actual electrode position (crosshair) is now to the right as the recording was made paracentrally in the left eye. The latencies are 15.9 and 16.5 ms for the OFF-responses and 17.4 and 16.9 ms for the ON-responses. OFF-responses again evolve in a stepwise fashion with small rise times, whereas ON-responses evolve more smoothly with longer rise times. Moreover, the rightmost ON-response has two ON-correlation peaks. We found such double-ON-peaks in 5–7 of 53 spike-RFs (9–13 %; the uncertainty is due to low signal-to-noise ratio in two cases).

**Figure 5:**
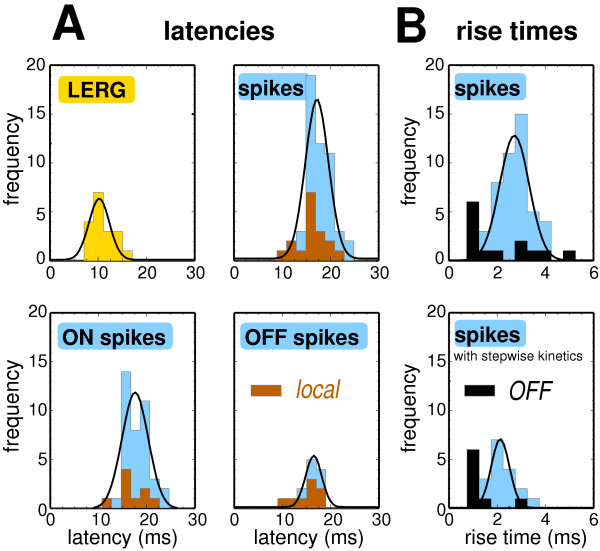
**Summary statistics for response latencies and rise times. A: **Latencies of LERG-RFs (yellow), pooled spike ON- and OFF-RFs, spike ON-RFs, and spike OFF-RFs (blue histograms with Gaussian fits, N = 18 LERG-RFs in two experiments, N = 53 spike-RFs in three experiments). Brown histograms of data restricted to *local *spike-RFs. **B: **Rise times for all spike-RFs (blue) and spike OFF-RFs (black). The upper plot shows pooled data for all three experiments. The lower plot shows data from the two experiments with stepwise response kinetics (experiments *142 *and *033*). Note here, that the rise times for spike OFF-responses are shorter than for spike ON-responses.

LERG responses generally had shorter latencies (11.9 ms ± 0.5 ms; N = 18 LERG-RFs in two experiments, standard error) when compared to spike-responses with *local *RFs (17.3 ms ± 0.7 ms, N = 17) or pooled *local and distal *RFs (18.6 ms ± 0.4 ms, N = 53). This is shown in Figure 5A, where we plotted frequency distributions for LERG (yellow), pooled ON- and OFF-spike, ON-spike, and OFF-spike RF latencies (blue). Note that the LERG latency distribution hardly overlaps the spike latency distributions. Superimposed on the latency distributions we plotted latency histograms for spike responses with *local *RFs (brown). Spike responses with *distal *RFs tended to peak later than those with *local *RFs most probably due to the additional axonal conduction time. Therefore, LERG latencies can best be compared with latencies of *local *spikes with latency differences of 5.4 ms ± 0.9 ms (two-tailed test, t(33) = 6.23, p < 10^-6^).

We further compared the response latencies for ON- and OFF-spikes. OFF-spike latencies were shorter (16.3 ms ± 1.0 ms, N = 8 *local *RFs) than ON-spike latencies (18.2 ms ± 1.0 ms, N = 9 *local *RFs). Latencies differed insignificantly by 1.9 ms ± 1.4 ms (t (15) = 1.35, p < 0.20). We estimated congruent ON- and OFF-spike RFs simultaneously with the same electrode at four recording positions. The mean latency difference in these cases was 2.4 ms ± 0.8 ms (1.6, 2.0, 2.5, 3.5 ms). The OFF-spike RFs temporally preceded the ON-spike RFs in all cases.

### Stepwise OFF-responses

In two of the three experiments with broadband recordings (*142*, *033*), the averaged time courses of spike OFF-responses to stimulation in the respective RF center appeared stepwise (Fig. 4). We therefore analyzed the steepness of the rising and falling phases of the time course for averaged ON and OFF responses, respectively (Fig. 5B). In the two experiments with stepwise signal kinetics, OFF-rise times were 1.6 ms ± 0.3 ms (N = 8) and ON rise times were 2.5 ms ± 0.1 ms (N = 15). The difference between the rise times was 1.0 ms ± 0.3 ms (t(21) = 3.63, p < 0.002). In the other experiment (*302*) OFF-responses appeared barely stepwise with rise times not significantly different (ON: 3.3 ms ± 0.1 ms (N = 25), OFF: 3.7 ms ± 0.5 ms (N = 5)).

## Discussion

### RFs of local electroretinograms

A general prerequisite for the generation of LERGs is the constructive superposition of extracellular field currents of individual neurons and glia cells [2,18]. Particularly retinal cells generating or relaying field currents in a roughly radial direction contribute to a field potential that can be registered with an epiretinal electrode. We recorded the locally generated field potentials (LERG) with the indifferent electrode placed in the vitreous body and the active electrode in close proximity to the inner limiting membrane (often somewhat impinging on it). This arrangement minimized the contribution of diffuse ERG from other sources [19,20].

The intact retina as used in our study provides several sources of graded local potentials, namely the receptor (outer nuclear layer), horizontal, bipolar, and to some extent the amacrine cells (inner nuclear layer) [1]. It is widely accepted that the graded potentials of the radially oriented receptors are responsible for the ERG's first negative deflection in response to a flash stimulus (*a-wave*, [3]). OFF-bipolar cells [21] and perhaps other more proximal cells [22] add to the shape of the a-wave. The a-wave can be observed with bright stimuli as used in our experiment. Under scotopic conditions the positive b-wave ERG component becomes more prominent. It originates mainly in ON-bipolar cells but is further influenced by OFF-center bipolar cells [23]. Thus, as the LERG originates in retinal processing that hierarchically precedes ganglion cell activity it makes sense that its latency is shorter than that of subsequent spike responses of ganglion cells as measured in this study. Given the polarity and short latencies (< 20 ms) we identify the first LERG component in our recordings as the ERG a-wave. We want to stress, however, that the LERG waveforms strongly depend on the electrode configuration and the stimulus used. Rodieck and Ford [18] argue that with small visual stimuli the distribution of extracellular currents from single cells must be considered as the lateral components do not cancel each other as with full-field stimuli. LERG responses to full-field and small visual stimuli can thus not easily be compared. Consequently, multi-focally evoked LERGs cannot be interpreted in a one-to-one manner in terms of ERG components measured with Ganzfeld stimuli [12,19].

All LERG-RF time courses showed negative initial peaks that were sometimes followed by a positive deflection (N = 34). Thus, field potentials were negative-going after ON-stimuli ("1" in the stimulus time course) and/or positive-going after OFF-stimuli ("-1" in the stimulus time course). This is typical of recordings with the recording electrode positioned intraretinally rather than in the vitreous humour [19,24].

The LERG yielded RFs that were always located at the position of the electrode tips projected to visual space. This strongly suggests that LERG are generated near the electrode tip. Interestingly, the localized nature of the LERG suggests that their RFs can be used as spatial reference points for the analysis of spike-RF positions. This can be very useful in experiments involving many retinal electrodes where the back-projection technique for the analysis of electrode positions would be cumbersome. However, a prerequisite is that the electrodes do not occlude the incoming light from the retina. The seven fiber electrodes in our setup were optically diffused as they were very thin and lying in a different focal plane than the photoreceptors. Their presence therefore had only minimal influence on the retinal light intensity distribution of the stimulus.

Another slow negative potential that can be invoked in the dark-adapted state with very dim light stimuli is the scotopic threshold response (STR) [25]. However, we used bright test stimuli that were not appropriate to elicit the STR. Moreover, STRs have longer latencies (more than 40 ms) than the a-wave evoked with brighter stimuli (less than 20 ms) [26]. The major (negative) response component that we observed clearly falls into the range for a-wave latencies. We therefore conclude that we did not misinterpret LERG a-wave/b-wave complexes with the STR.

We can further exclude that LERGs are merely artifacts due to impulse responses of the hardware filter to large action potentials because firstly, due to the causality of the used filters, LERG signals then needed to *follow *the spikes, instead LERG signals have shorter latencies than spikes. Secondly, LERG-RFs would have to be located at the same positions as the RFs of (large) spikes, which often was not the case.

### RFs of single unit spikes

The RFs of single unit spikes simultaneously registered by the same electrode were either located at the site of the corresponding LERG-RF of this electrode (*local *RFs, 43 % of 61 spike-RFs) or tended to line up in a way that correlated well with the estimated fiber trajectories (*distal *RFs, 57 % of 61 spike-RFs). This strongly indicates that displaced spike-RFs result from the recording of axonal spikes originating at more distal locations along the fiber bundle and passing the recording electrode *en route *to the optic disk. It is not surprising that we registered axonal spikes since axons were in much closer proximity to the recording electrodes than the ganglion cell somata in situations where the electrode tip just touched the inner limiting membrane [27]. In addition to that we need to recall that the axonal trajectory [8] and layering [9,10] is highly organized in a way that the distance between the recording electrode and an axon correlates with the retinal eccentricity of the axon's origin. The amplitude of a recorded spike should therefore correlate with the retinal distance to its site of initiation (the ganglion cell soma). However, we could not find a correlation like this, possibly due to the uncertainty of the exact retinal recording depth. Moreover, axons originating in more peripheral retinal sites have larger calibers and hence produce larger voltage drops at the recording electrode than axons originating from peripapillary locations.

Given the limited angular extent of the visual stimulus in our setup (max. 17.5° monitor diagonal), it was a challenge to map *distal *RFs which could easily be separated by ten or more degrees of visual angle. Reducing the distance between eye and stimulus monitor increased the viewing angle and therefore the probability of mapping the RFs but at the expense of angular resolution. Additional RFs beyond the monitor's stimulation field were manually confirmed on the *distal *but not the *proximal *side of the LERG-RFs in all experiments. On average, we found 1.8 spike-RFs per retinal recording electrode. This number surely gives a lower limit as additional, more *distal *RFs were not detected, and more sophisticated event separation algorithms might filter out even more single neuronal units (e.g., [28]). Higher spatial stimulus resolutions can probably help to further separate neurons with strongly overlapping RFs. It has to be considered, though, that stimulus energy decreases with smaller pixels. The signal-to-noise ratio of the evoked responses will therefore decrease due to a lower spike activation probability.

### Temporal aspects of retinal RFs

Spikes and LERGs simultaneously registered with the same electrode often had congruent RFs (*local *RFs). In these cases, however, LERG-RFs appeared at shorter latencies than spike-RFs. Since for *local *RFs optimal stimulation was done with the same stimulus pixels (i.e., in the RF center), we can exclude stimulus timing issues as an explanation for the latency difference.

Chichilnisky & Kalmar [13] performed *in vitro *multi-electrode recordings from flat mounts of macaque retinae and detected functional asymmetries in ON and OFF ganglion cells (probably parasol (magno) cells, comparable to cat Y-cells) including response kinetics. In particular they found that ON cells had a 10–20% shorter time-to-peak, trough and zero-crossing in the biphasic temporal impulse response than OFF cells. However, they also note that the response latency which they estimated as the time to 5% of the peak response was on average 1–2 ms shorter for OFF cells than for ON cells. The latter is in good accord with our estimation of a 1.9 ms latency difference between ON and OFF responses (time to 70% of peak response). The reason why we did not analyze the response peak times in more detail is that we found the OFF peaks to exhibit stepwise time courses that deemed it inappropriate to define a peak time because of their flat peak plateau (e.g., Fig. 4). Along with Chichilnisky & Kalmar [13] we assume that the biochemical steps involved in the generation of the ON-bipolar response yield a longer latency for ganglion cell spike initiation than the directly gated ionic currents underlying the OFF-bipolar response. This is because ON-center bipolar cells have metabotropic glutamate receptors that mediate a (time-consuming) response polarity reversal leading to membrane depolarization. On the other hand OFF-center bipolar cells have ionotropic glutamate receptors and respond to light like the photoreceptors with a membrane hyperpolarization which is less time consuming [29,30].

This asymmetry in the retinal ON/OFF pathways may also be accountable for the stepwise time course of cross-correlation functions between stimulus and OFF-responses in two of the three experiments with broadband recordings (Fig. 4). Here, OFF rise times were about 0.9 ms shorter than ON rise times (1.6 and 2.5 ms, respectively). This observation did not depend on the animals' medication nor the electrode recording depth in the retina since we saw smooth ON- and stepwise OFF-responses among simultaneously recorded single unit spikes from the same electrode (Fig. 4). As probably less retinal stages are involved in the initiation of OFF-center ganglion cell spikes the average spike onset time might be more reliably encoded and consequently yields a steeper rise in the cross-correlation function between stimulus and OFF-response. OFF-responses appeared barely stepwise in one other experiment (*302*). Here, rise times for ON- and OFF-responses were similar and both longer than for stepwise OFF responses. From the limited number of observations we cannot deduce why OFF-responses were stepwise in some recordings and not in others.

Based on the knowledge of spike responses recorded by the same electrode with *local *and *distal *RFs one can in principle deduce the spike conduction time as the difference of their response latencies. This is only true for RF-pairs with the same polarity as we argued that the signal transduction kinetics for ON- and OFF-responses differ and thus add different temporal delays to the overall signal latency. Response latencies varied considerably (Fig. 5A), though, sometimes leading to a *distal *RF temporally preceding a *local *one recorded with the same electrode despite the extra conduction time. This may indicate that single unit spike responses are sometimes recorded from different types retinal ganglion cells or amacrine cells. For example, Cleland and Levick [31] found that latencies differ for X and Y cells and depend on the retinal eccentricity. We did therefore not correct the latencies of responses with *distal *RFs for the additional conduction time. With a spike conduction velocity of about 1 m/s [32], the observed retinal RF separations of 0–2 mm correspond to about 0–2 ms extra conduction time. This is smaller than the latency difference between LERG and spike responses (6.7 ms, cf. *Results*) and further indicates that the source of the LERG response to visual stimulation must be precedent to spike initiation.

### Consequences for the design of an epiretinal implant

Our results may have important implications for the design of epiretinal implants that aim at substituting retinal function by eliciting localized visual percepts ("phosphenes") via focal electrical stimulation [15,16,33]. Rather simplifying we assume that a similar group of retinal neurons may be stimulated as well as recorded from by the same epiretinal microelectrode. We thus imply the reciprocity of some properties of epiretinal recording and stimulation. This is reasonable as the electrode-retina interface is the same in both cases. The electrical field seen by a recording electrode is spatially similar to (but not the same as) the electrical stimulation field seen by retinal neurons since both depend on the distance between the electrode and the neuronal structure. Since we used microelectrodes with relatively large tips, the probability of recordings of spikes from fibers with large diameter (Y-cells) was high [27]. As the fibers of large diameter also have lower stimulation thresholds [34] it seems probable that the fibers preferentially recorded from were also those that would be preferentially activated by a short impulse via that electrode. However, neuronal elements can be stimulated indirectly via synapses of other retinal neurons. Moreover, the spatial structure of the neuronal sources and sinks, i.e., the shape of the individual cell recorded from must be known to predict which parts of the cell can be stimulated [34]. Also, degenerating retinae found in blind patients suffering from macular degeneration or retinitis pigmentosa undergo an extensive retinal remodelling that can further complicate the issue of controlled stimulation of retinal targets [35].

Our data demonstrate multi-site epiretinal RFs suggesting that epiretinal electrical stimuli might unspecifically activate retinal axons that originate in an elongated area of the retina distal to the stimulation site, potentially resulting in multi-site or elongated visual percepts. One way of dealing with multiple axonal stimulation would be to reduce the distance between electrode and ganglion cell somata by advancing small-tipped cone electrodes to the cell body layer. This would increase the probability of activating ganglion cell somata rather than axons. One could in principle test RFs of neurons from "quasi-intraretinal" recordings for contributions of multiple sources and adjust the recording depth until only a local component remains. We therefore tested the retinal RFs' dependence on the recording depth in one pilot measurement: We measured four LERG-RFs and one corresponding spike-RF. The LERG-RFs exhibited a slight latency decrease with penetration depth (appr. 5–10 ms/100 μm). Additionally, the polarity of LERG-RFs abruptly reversed at a certain recording depth (appr. 250 μm from the inner limiting membrane) which was accompanied by an increase in LERG-RF size. This is a typical situation if the choroidal side of Bruch's membrane is penetrated [19]. In contrast to the LERG, the latency of the averaged spike-response did not change with recording depth. Rather, the signal-to-noise ratio of the spike-RF in this example degraded with recording depth so that the spike-RF was not recognizable from background noise when the LERG-RF polarity reversal was complete. All of these effects were reversible when the recording depth was reduced. We assume that the suppression of the spike-RF indicates that quasi-intraretinal electrical stimuli can avoid axonal activation and be rather localized. It must be stressed that these results are from few recordings in one cat only which need to be further investigated in subsequent measurements.

McIntyre & Grill [36] approached the issue of the selectivity of neuronal activation studying a computer-based integrated field-neuron model. They found that the stimulus waveform can be a means of selective activation of targeted neuronal populations. Asymmetrical stimulus waveforms with a long-duration low-amplitude prepulse followed a shorter and high-amplitude compensating main stimulus pulse were among the most effective in selectively activating local cells rather than fibers of passage. If only one, displaced, perceived phosphene remained, this could be compensated for by an electronic preprocessor that maps the camera input to the retinal electrode grid [16].

Whether or not epiretinal electrical stimulation yields multi-site visual sensations must ultimately be tested in alert subjects that can report evoked visual percepts. Very promising data have been collected by Humayun and coworkers [37] who implanted an 4×4 epiretinal electrode array with disk shaped electrodes of 520 μm diameter into a blind subject's eye. Interestingly, they did not report displaced phosphene percepts and argue that a biphasic current pulse of 1 ms/phase and 1 ms interphase delay can activate a local pool of bipolar cells. As their stimulation electrodes were rather large, it remains an open issue whether retinotopic phosphene percepts can be guaranteed for much more densely packed electrodes as they would be needed for useful vision [38,39]. Indeed, contrary to Humayun and coworkers, Rizzo et al. [40] often observed elongated or multi-site visual percepts in human patients when they electrically stimulated their patients with single epiretinal electrodes. When stimulated with multiple electrodes, the perception of either elongated or multi-site phosphenes depended on the stimulation array's orientation relative to the ganglion cell axon trajectory underneath the electrodes. This supports our hypothesis that epiretinal electrical stimuli can lead to ambiguous visual percepts and that the analysis of epiretinal visual RFs can help to optimize the design of an epiretinal implant for substituting basic visual function.

## Conclusion

Our data demonstrate the feasibility of obtaining highly specific receptive field data from different neural sources by epiretinal broadband recordings with microelectrodes. We could separate distinct retinal signals from the epiretinal broadband recordings which enabled us to study the RF properties of simultaneously recorded signal components from different retinal sources. The caveats that come with recordings from the retinal surface, i.e., the likelihood of mixed contributions from different retinal neurons, may also have implications for the design of an epiretinal visual implant.

## Methods

### Anesthesia, surgery, and animal care

For semichronic preparation adult cats (N = 4; 3.0 to 4.5 kg) received atropine sulphate (0.03 – 0.04 mg/kg) to reduce salivation. Anaesthesia was induced by intramuscular injection of a mixture of ketamine hydrochloride (Ketanest, 10–15 mg/kg) and xylazine hydrochloride (Rompun, 0.7 – 1.0 mg/kg). After orotracheal intubation, anaesthesia was maintained by ventilation with N_2_O / O_2 _(70 % / 30 %) and isofluorane (0.5 – 1.5 %). The level of anaesthesia was continuously controlled by monitoring the rectal temperature (38°C), endexpiratory CO_2 _(3.8 – 4.2 %), ECG, EEG, and muscle reflexes. For head fixation we implanted two bolts in cavities of the forehead by dental acrylic cement that enabled positioning in a standard Horsley-Clark support without pressure points typically induced by the use of ear bars. The operated eye was stabilized with the help of the electrode array and two sutures that were pierced through the conjunctiva and fixed to the Horsley-Clark support. Refraction of the eyes was corrected for the eye-to-monitor distance of 1.3 m using contact lenses. Besides during the ophthalmic surgery it was not necessary to give further analgesia between recording sessions as was assured by monitoring the cat's vital functions. One of the four cats was not sacrificed at the end of the recording sessions. In this case, sclera and conjunctiva were closed with polyglactin sutures and the animal prophylactically received penicillin. This semi-chronic preparation enabled repeated experiments in the same eye after allowing 2–3 weeks for convalescence. At the end of the other three experiments the animals were deeply anesthetized with isofluorane (3 %) followed by an intravenous injection of a lethal dose of T61 (Hoechst Roussel Vet, 2 ml). All animal experiments were conducted in accordance with the German animal welfare law, the guidelines of the European Community Council Directives (86/609/EEC) and the NIH Principles of Laboratory Animal Care (Publication 86-23, revised 1985).

### Multiple microelectrode retinal recordings

Epiretinal recording was performed with fiber microelectrodes [41] with cone-shaped tips of 20–25 μm diameter and 200–500 k impedance at 1 kHz. We used a 7-electrode array (modified from the manipulator of Eckhorn & Thomas [42]) to insert the electrodes through a 1.1 mm scleral opening approximately 4 mm posterior to the temporal limbus. Electrode separations were either 0.3 mm (N = 2) or 0.25 mm (N = 2). In the latter case, three additional stainless steel capillaries similar to those guiding the electrodes were inserted through the same scleral opening as a means for adjusting the inner eye pressure via an elevated support of Ringer's solution (experiment *302 *and *033*). The electrodes were hexagonally arranged to provide for a two-dimensional recording and to minimize the scleral opening by a dense packing of electrodes (0.9 mm diameter of total bundle of guide capillaries). Retinal electrodes could be individually moved in axial direction under computer control. This allowed for precise epiretinal positioning of the electrode tips under visual inspection with an ophthalmoscope. Additionally, the whole electrode manipulator could be spherically moved around the electrodes' point of insertion into the eye so that a wide range of retinal positions could be approached without mechanical stress to the eye [43]. The tips of the electrodes were positioned 4° to 9° paracentrally with very low pressure on the retina. This resulted in a slight dimpling of the retinal surface that could precisely be adjusted under visual inspection with an ophthalmoscope and double-checked by listening to the onset of spike activity via an audio monitor. The epiretinal positions of the electrode tips were marked on a tangent screen with a custom-built back-projection device [44].

### Data recording and pre-processing

Vital functions of the cat, including ECG, EEG, body temperature, and expiratory CO_2 _were continuously monitored during the experiments. Data were recorded and considered for analysis only when vital functions were normal and stable. Between the measurement sessions, all electrodes were individually retreated to a safe distance in the vitreous body, the electrode array was moved to another retinal site, and the electrodes individually positioned on the retinal surface again. This took at least 30 minutes. The typical interval between sessions was 60 minutes.

In our first experiment (*140*) we hardware-filtered the retinal signals to obtain local electroretinograms (LERG: 1–140 Hz, -3 dB at 12 dB/oct) and multi unit activity (MUA: 0.5–10 kHz, full wave rectified, then low pass filtered to 1–140 Hz, -3 dB at 30 dB/oct). These filtered signals were then sampled at 500 Hz (CED 1401plus, Cambridge Electronic Design) and stored for offline data analysis. Since we wanted to separate single unit spike trains contained in the raw signal we recorded broadband signals (1–4000 Hz) at 20 kHz sampling rate (Multi Channel Systems, Tübingen) in all subsequent experiments (*142*, *302*, *033*). For the sake of comparison with our first data set (*140*), LERGs were then extracted off-line by convolving the broadband signals with the impulse response of our laboratory hardware filter. Using an amplitude thresholding approach enabled us to extract spikes from high-pass filtered (0.5–1 kHz) broadband data: First, local signal peaks of a certain range of widths were detected by the analysis of the first and second derivatives of the signal time series. In a second step, the peaks were sorted into ranges of amplitudes and stored into separate event data files. However, it cannot be ruled out that one sorted spike population comprised more than one spiking neuron.

### Visual stimulation and receptive field evaluations

Monocular RFs were qualitatively evaluated with a hand-held projector by stimulating with an adjustable light bar on a tangent screen 1.3 m in front of the cat. This distance also left enough room to quickly access the eyes for medical treatments between the recording sessions. For the quantitative characterization of the position, size, subfields, and orientation of RFs we used an automated mapping technique in which the luminance of a computer monitor was randomly spatio-temporally modulated covering the RF positions of all retinal recording locations simultaneously (28×28 grid of 0.45°×0.45° stimulus pixels, total range 12.5°×12.5° visual angle, maximum contrast of a computer monitor at 101 Hz frame rate in a dark room, the luminance of the white pixels was 75 cd/m^2 ^and the luminance of the black pixels 0.1 cd/m^2^). The multi-focal pseudo-random stimuli were based on binary m-sequences with 4095 steps [45,46]. These were generated using a feedback shift register algorithm [47,48] with subsequent testing for m-sequence properties. On average 50 % of all grid positions were bright in each stimulus frame and each pixel was bright in 50 % of the stimulus frames. Each stimulus time course was corrected for its temporal delay due to the row-by-row cathode ray scanning of the computer monitor. The spatio-temporal RFs were determined by cross-correlation among the binary luminance time course of each stimulus pixel ("1" for bright and "-1" for dark stimuli) and the retinal LERG- and spike-responses, respectively (Fig. 1B). Thus, a positive cross-correlation can either result from a signal increase in response to a bright stimulus or a signal decrease in response to a dark stimulus. Likewise, a negative cross-correlation can result from signal decrease in response to a bright stimulus or signal increase in response to a dark stimulus. Each multi-focal m-sequence was presented 45 times (total duration 30 min, experiment *140*) or 15 times (total duration 10 min, experiments *302*, *033*, *142*) to improve averaged cross-correlations between stimulus and response. The temporal resolution of the stimulus was limited by the monitor frame rate (9.9 ms frame duration). We therefore resampled the stimulus and signal time courses at 50 times the stimulus' temporal resolution (i.e., 0.2 ms) prior to calculating the cross-correlation functions in order to provide for sufficient temporal interpolation.

In a further step we plotted color-coded 2-dimensional maps of the correlation values between each stimulus time course and retinal response at certain temporal delays (Fig. 1C). For ease of interpretation we eightfold interpolated the correlation maps by zero-padding in the frequency domain [49]. Color coding was adjusted for each signal separately in such a way that the lowest negative correlation value was plotted in dark blue and the highest positive correlation value in light yellow. A sequence of such correlation maps for successive temporal delays ("RF time course") can thus be interpreted as the averaged retinal response amplitude or spike probability over time for all visual stimulus positions or as the mean visual stimulus given before a retinal response is recorded [50].

With our multi-electrode approach we were able to map up to seven epiretinal sites at once. From each electrode signal we separated one LERG signal and often several single unit spike trains which were then tested for the center position, time course, and polarity of their RFs (ON- or OFF-center). The RF center positions were estimated from the peak maximum of 2-dimensional RF maps (low-pass-filtered to half of the stimulus grid's spatial frequency). Once the RF center was located, we analyzed the time course of the RF as given by the cross-correlation function among the time course of the optimal stimulus pixel and the retinal signal. In order to improve the signal-to-noise ratio of the estimated RF time course, we averaged the time courses for the optimal stimulus pixel and its adjacent eight stimulus pixels. This was possible since most RFs extended over more than one stimulus pixel width. Visual RF center positions were compared by superposition in a common frame of reference in visual and retinal space (Fig. 3).

The polarity of a RF (ON- vs OFF-center neuron) was defined as the polarity of the first major signal deflection in the cross-correlation function. Moreover, we calculated the time elapsed from the stimulus onset to 70% of the RF peak amplitude as a measure for the retinal response latency. We also analyzed the steepness of the rising and falling phase of the time course for averaged ON and OFF responses, respectively. As a reliable measure for this we used the signal rise time from 30% to 70% of the RF peak amplitude.

The calculated RF center positions were compared to the back-projected electrode tip positions. This way we were able to check for any deviations of functional RF positions from the geometrically expected ones. Based on the work of Stone & Holländer [8], we further identified the approximate trajectory of ganglion cell axon bundles in the vicinity of the electrode position by back-projecting the optic disk and pattern of major retinal blood vessels. All data analyses were performed using the program language IDL (Research Systems Inc., Boulder, CO, U.S.A.).

## Authors' contributions

MW performed the experiments, analyzed the data, and drafted the manuscript. RE contributed to experiment conception, design, and the critical revision of the article.
